# Biodegradation of Emerging Contaminants Controlled by Biological and Chemical Factors

**DOI:** 10.3390/microorganisms13102354

**Published:** 2025-10-14

**Authors:** Avela Mqambalala, Maleke Maleke, Jorge R. Osman, Julio Castillo Hernandez

**Affiliations:** 1 Department of Microbiology and Biochemistry, University of the Free State, Bloemfontein 9300, South Africa; avelamqambalala@gmail.com; 2 Department of Life Sciences, Central University of Technology, Bloemfontein 9300, South Africa; mmaleke@cut.ac.za; 3 Instituto de Geología Económica Aplicadà, Universidad de Concepción, Concepción 4070386, Chile; osman.jorge@gmail.com; 4 Department of Integrated Science, Campus El Carmen, University of Huelva, 21007 Huelva, Spain

**Keywords:** emerging contaminants, biodegradation, genes, metabolism, co-metabolism, transformation products

## Abstract

Emerging contaminants (ECs) are organic compounds, including pharmaceuticals, personal care products, pesticides, and other chemicals, that are linked to harmful effects on aquatic environments. Indigenous microorganisms often act as natural barriers by breaking down these contaminants into less harmful substances. However, not all biological processes result in the complete biodegradation of ECs, and specific conditions must be met for this to occur. These conditions are influenced by chemical and biological factors such as seasonal variations, oxygen availability, nutrient levels, ECs concentrations, and the types of microorganisms present in the aquatic environment, all of which can either enhance or inhibit ECs biodegradation. This review provides a thorough examination of the chemical properties and factors that influence the fate of ECs in aquatic environments, discussing the impact of these factors on microbial degradation of ECs through metabolic and co-metabolic processes. Finally, this review emphasizes the importance of integrating interdisciplinary studies that consider diverse key factors to offer a more holistic and accurate understanding of ECs’ biodegradation in aquatic systems.

## 1. Introduction

**Figure 1:**
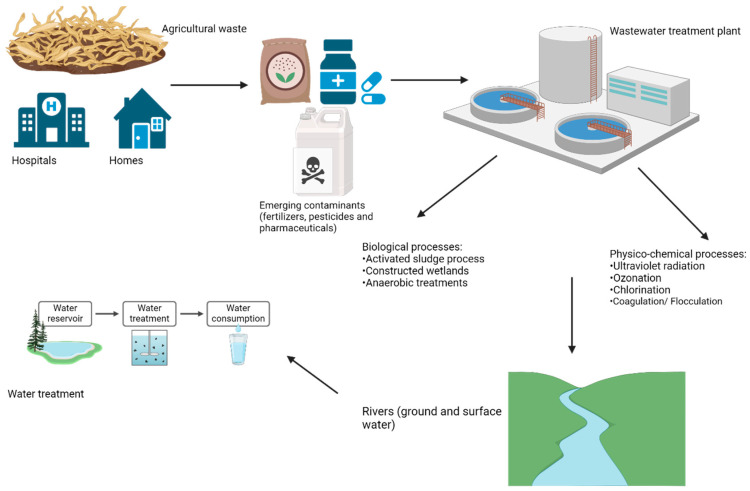
Sources and fate of emerging contaminants, including methods of degradation in the environment.

Emerging contaminants (ECs) are naturally or synthetically produced organic compounds that are not currently environmentally regulated but have adverse effects on human health and ecosystems [1,2,3]. These compounds can be found in water and soil and include agricultural chemicals, industrial compounds or by-products, personal care products, pharmaceuticals, tertiary products, nanomaterials, and their metabolites. These compounds can reach the environment via agricultural, laboratory, domestic, and hospital wastewaters, industrial discharge, street run-off, precipitation, landfill leaching, and sewage treatment effluent (Figure 1) [1,3]. The fate (including degradation) and the transport of these emerging contaminants depend on their physicochemical properties (e.g., solubility, pH, mineral concentrations), which are affected by the surrounding environment and can be beneficial or toxic to the aquatic environment. They occur in trace-level concentrations (ng/L or µg/L) and can inhibit microorganisms (e.g., microalgae) that contribute to the homeostasis and functionality of aquatic ecosystems [4,5]. The frequent interaction of microorganisms with ECs, such as antibiotics or disinfectants, has also been linked with the induction and development of antimicrobial resistance [6,7,8]. Even though antimicrobial resistance can have direct consequences to human health, bioaccumulation and biomagnification should not be overlooked. These two crucial processes intensify the spread of ECs throughout the food chain. For instance, the pesticide di-chlorodiphenyltrichloroethane (DDT) can be bioaccumulated or biomagnified in aquatic animals (e.g., fish and algae) which are then, consumed by humans [9].

Indigenous microorganisms in aquatic environments have demonstrated the ability to break down high molecular weight compounds, such as ECs, into intermediate compounds, which can either be harmless or harmful [10]. Biological degradation of ECs has been extensively reported. In aquatic ecosystems, microorganisms such as bacteria (*Arthrobacter*, *Pseudomonas, Sphingomonas*, among others) [11,12,13,14] and fungi (*Aspergillus*, *Fusarium*, and *Penicillium*) [15,16,17] seem to break down ECs to assimilate C, N, or S compounds into their metabolisms [10,18]. Some studies have indicated that mixed microbial cultures can enhance degradation efficiency [19,20,21]. The potential for co-metabolism, where one microbial species can degrade emerging contaminants as secondary substrates that are not needed for growth, might be crucial to catalyze the degradation of ECs. However, the mechanisms underlying these interactions are not well understood. Likewise, the concentration required for ECs to trigger microbial metabolic activities remains uncertain. Given that the concentrations of ECs range from µg/L to ng/L, and those other organic and inorganic compounds often predominate in solution, EC biodegradation through co-metabolism seems more feasible in natural aquatic environments. Co-metabolism allows microorganisms to degrade contaminants at low concentrations by utilizing other primary substrates for growth, while simultaneously transforming ECs without directly benefiting from their breakdown [22,23,24]. Therefore, indigenous microorganisms seem to play a role as natural barriers, breaking down ECs.

The efficiency of ECs’ degradation through metabolism and/or co-metabolism could be affected by biological and chemical factors, whose processes remain unclear [25,26,27]. Biological and chemical factors such as nutrient availability and oxygen fluctuations, seasonal variation, ECs concentrations, and the type of microorganisms can significantly affect the microbial composition and functionality associated with ECs’ degradation [28,29]. Mahmoudi et al. (2013) emphasized the role of biological and chemical factors in biodegradation rates of spilled oil and noted that these factors interact with microbial community dynamics and functionality [28]. This was based on a study by Leahy and Colwell, which indicated that factors such as oxygen, nutrient availability, and pH had an effect on the microbial biodegradation of petroleum hydrocarbons [30]. Oxygen was indicated to be essential for the microbial oxidation of hydrocarbons by oxygenases [30]. Nutrient availability of nitrogen and phosphorus and extremes of pH were seen to limit the microbial biodegradation of the hydrocarbons [30]. Atlas also stated that seasonal variation has an effect on the microbial composition, which will affect the biodegradation rates [31]. Atlas showed that higher hydrocarbon metabolism was higher in winter as compared with other seasons [31]. On the other hand, Liang et al. (2010) highlighted the need for more detailed investigations into the dynamics of specific genes involved in the degradation of emerging contaminants [32]. Indeed, understanding the functional gene repertoire is crucial for predicting the metabolic capabilities of microbial communities and their resilience to the contaminant. This review provides a comprehensive analysis of the chemical properties and fate of ECs in aquatic environments, with a special emphasis on those originating from South Africa. South Africa has unique agricultural, industrial and pharmaceutical usage patterns that ultimately affect EC concentrations. Consequently, significant amounts of ECs and their metabolites are excreted and enter the municipal wastewater systems. Because conventional wastewater treatment plants are not designed to effectively remove these ECs, this leads to their persistence in effluents and eventual release into surface waters. This poses potential ecotoxicological risks to aquatic ecosystems and may contribute to the development of antimicrobial-resistant strains. Therefore, the review assesses the potential of microbial biodegradation of ECs through metabolic and co-metabolic processes, highlighting the influence of biological and chemical factors on their effectiveness.

## 2. Chemical Characteristics of Common Emerging Contaminants

The fate and behavior of ECs in aquatic systems depend on their chemical and physical properties [33,34,35,36]. These properties may have an effect on their bioavailability to microorganisms, which in turn will affect their biodegradation [27,35,37]. For example, ECs with high adsorption potential are adsorbed into the sewage sludge, which decreases their bioavailability [37,38]. Generally, the chemical properties are determined by the structure and the atoms present in the ECs [39]. These chemical characteristics allow ECs to persist in the aquatic environment. Moreover, factors such as EC solubility and/or lipid solubility can also influence their persistence in the environment, which is affected by the structure and arrangement of the molecules that make up the ECs [39]. The chemical structures of ECs can also have an effect on their biodegradation [35,36]. For example, the degree of chlorination in polychlorinated biophenyls increases their persistence in the environment, which decreases their biodegradation [36]. This section focuses on the chemical characteristics of different classes of ECs and their overall effect on their fate and behavior.

### 2.1. Pharmaceutical and Personal Care Products

Pharmaceutical and personal care products are usually polar, non-volatile, and heat sensitive, with pH varying between acidic and basic and a high water solubility in the aqueous phase, while the ones with low solubility stay insoluble with solid matter found in wastewater [40]. Typically, pharmaceuticals include compounds in anti-inflammatories and analgesics such as acetaminophen (paracetamol), Ibuprofen, and diclofenac. They also include antidepressants such as benzodiazepines, anti-epileptics such as carbamazepine, lipid-lowering drugs such as fibrates, beta-blockers (atenolol), anti-ulcer drugs and antihistamines (ranitidine), and antibiotics (e.g., sulfamethoxazole) [41,42]. These compounds have a molecular weight of less than 500 Da and are formed by large and chemically complex molecules that differ in molecular weight, structure, functionality, and shape (Table 1). These compounds have polar regions with more than one ionizable group, and the ionization of these groups depends on the medium’s pH [42]. Pharmaceutical compounds can also stay long in the aquatic environment due to their highly stable chemical structures [41,42,43].

Personal care products include compounds such as salicylic acid (analgesic), triclosan (antimicrobial), galactosidase (fragrances), tonalite (fragrances), climbazole (fungicidal), ethinylestradiol (hormone), sodium lauryl sulfate (anionic surfactant), and sodium dehydrogenate (preservative) (Table 2). The compounds have lipophilic properties, which allow them to remain in aquatic environments for long periods [45,46]. These properties have also been linked to bioaccumulation [47,48,49], ecological impacts on the environment (bioaccumulation) and their biodegradation [50,51,52].

### 2.2. Per- and Poly-Fluoroalkyl Substances

Per- and poly-fluoroalkyl substances are fluorinated aliphatic compounds that are highly stable in the environment and often exhibit low to moderate water solubility (Table 3). They include several groups such as per-fluoroalkyl carboxylic acids (e.g., trifluoroacetic acid, perfluoropropanic acid, perfluorobutanoic acids), per-fluoroalkyl sulphonic acids (e.g., perfluorobutanoic sulphonic acid, perfluorooctanoic sulphonic acid, perfluorohexane sulphonic acid), per-fluoroalkyl phosphonic acids (e.g., perfluorohexane phosphonic acid, perfluorooctane phosphonic acid, perfluorodectane phosphonic acid), per-fluoroalkyl iodides (e.g., perfluorohexyl iodide, perfluorooctyl iodide, perfluorodecyl iodide), and perfluoroether carboxylic acids (e.g., Hexafluoropropylene oxide trimer acid) [53,54,55].

### 2.3. Pesticides

Pesticides (Table 4) include a variety of compounds such as atrazine, Isoproturon, lindane, dichlorophenyltrichloroethane (DDT), and metolachlor, which all form part of commonly used herbicides and insecticides [56]. Their chemical composition is rather complex and consists of natural and synthetic organic and inorganic compounds [57]. Based on the chemical compositions, they are classified into organochlorines, organophosphates, carbamates, pyrethrins, and pyrethroids [57]. Organochlorides are organic compounds with five or more chlorine atoms attached to them. These pesticides have a low solubility in water (0.005–8.5 mg/L), which allows them to persist in the environment, thereby making them highly toxic [58]. On the other hand, organophosphates are hydrocarbon compounds derived from phosphoric acid that contain one or more phosphorus atoms in their molecule [58]. The basic structure consists of a phosphate group, and the substitutes can either be methyl or ethyl groups, while the one oxygen atom can be replaced by sulfur atoms; the other oxygen can be attached to any other atom [59]. They have a low persistence in the environment due to their high solubility in water (1.5–23,800 mg/L) and are easily hydrolyzed, which allows them to be readily biodegradable [57,58,59]. Carbamates are derived from carbamic acid and contain an alcohol, methyl, and a hydrogen group [57,58,59]. These compounds also have a very high water solubility (120–280,000 mg/L), which allows them to be readily biodegradable in the natural environment [57,58]. Pyrethrins are naturally occurring organic compounds that contain five esters, with asymmetric carbons and double bonds in the alcohol and acid moieties that are formed from *trans* acids and *cis* alcohols [58]. Pyrethroids are synthetic derivatives of natural pyrethrins. They differ from the pyrethrins, as they contain a phenoxy group and halogens with a low water solubility (<0.002 mg/L) but are easily degradable by photodegradation [57,58,59]. The physicochemical parameters such as solubility, logKow, and pKa (Table 1 and Table 2) are regarded as essential components in predicting the behavior of personal and personal care products in the aquatic environment [60].

## 3. Sources and Fate of Emerging Contaminants

Emerging contaminants enter the aquatic environment via point sources [1,41,61,62]. Principal sources include drugs excreted that are disposed of into the domestic sewage system, leaching from landfills, hospital effluents, runoff from animal husbandry and aquaculture sites, drug manufacturing companies, and wastewater and sewage treatment plants [63]. Other sources of ECs, such as agricultural run-off, are responsible for transporting herbicides from agricultural land into surface water, which is treated and used as drinking water [1,63,64,65]. For instance, high concentrations of ECs such as atrazine (1237 ng/L) and terbuthylazine (1969 ng/L) have been detected in surface, ground, and wastewater (Table 5) from South Africa in the Free State, Gauteng, and North West Province [64,66]. Urine-diverted dry toilets have also been identified as one of the major sources of ECs in South Africa. Emerging contaminants that have been detected from these toilets include atenolol (25,900 ng/L) [67] and sulfamethoxazole (34.5 µg/L), which are associated with their consumption by HIV-positive patients for antibacterial infection and prophylaxis for long-term consumption [64,68]. The urine from toilets with high EC concentrations is directly disposed of into soak-way pits, which can leach into groundwater [64,67]. Anti-retroviral compounds used for HIV treatment have also been detected in ground, surface, drinking, and wastewater. Maximum concentrations of ECs detected include nevirapine (1480 ng/L), zidovudine (973 ng/L), lamivudine (242 ng/L), and stavudine (778 ng/L) in different parts of South Africa. Wood and co. reported that nevirapine (139 ng/L) and zidovudine (339 ng/L) were identified as the most abundant in Hartbeespoort Dam, South Africa (Table 5) [64,66,69].

In addition, solid and semi-solid wastes are usually dumped into landfills, which may contain ECs from unwanted medications, soft drinks, and other personal care products [70]. The improper management of the landfill leachate can introduce ECs into freshwater sources. The disposal of landfill leachates can lead to an increase in ECs in wastewater treatment plants, eventually reaching drinking water [64,70]. For instance, Polybrominated diphenyl ether has been detected in landfill leachates from landfills in Cape Town, South Africa [64].

In natural environments, adsorption can affect the fate of ECs by influencing their movement, plant uptake, and bioavailability. In such instances, the ECs can remain in soil due to their physicochemical properties such as the molecular structure, water solubility, and hydrophobicity [70,71]. The hydrophobicity can result in low solubility, which will allow the EC to interact with the soil over time, forming stronger bonds, which decrease the bioavailability of the EC, decreasing its exposure to biotransformation by microorganisms [72,73], but they may be toxic to soil living organisms. Lower bioavailability can also result in a lower intake of the EC by organisms, reducing their adverse effects [74]. ECs can also end up in the atmosphere via volatilization from surface water. They combine with airborne particles, which may interact with air-breathing organisms [65,70,75]. Pesticides such as atrazine and terbuthylazine have been detected in precipitation entering the atmosphere through their application as sprays. ECs can also flow into rivers via filtration at different rates, which depend on the sorption affinity to suspended solids or sediments and their resistance to chemical or biological degradation [65,70].

Wastewater treatment plants can act as primary inhibitors against ECs, preventing them from entering the environment. The ECs can undergo chemical and biological treatments in sewage treatment plants and wastewater treatment plants, and the fate of most organic ECs is dependent on their degree of natural attenuation [63]. These ECs eventually get biodegraded into different metabolites, which can be toxic, or they can remain unprocessed for long periods [65,70]. Spontaneous biological degradation can be facilitated by microorganisms that are naturally present in sewage treatment plants, found naturally in suspended soils/ sediments via hydrolysis, oxidation, and reduction [65]. Wastewater treatment plants can remove some organic compounds, which contain carbon, nitrogen, and phosphorus. However, they are not designed to remove persistent ECs [70,76]. For instance, removal efficiency for many of the widely used pharmaceuticals is as low as 10% [77]. During primary treatment, hydrophobic ECs can be absorbed into primary sludge, which partially removes these ECs from the dissolved state after treatment [76]. In secondary treatment, emerging organic contaminants can be transformed via aerobic and anaerobic microbial metabolisms. At this stage, ECs can be biodegraded to varying extents. Incomplete degradation may occur due to a lack of functional genes or insufficient microbial diversity in the environment [70,76]. The microorganisms can perform biological biodegradation using metabolic and co-metabolic processes. Based on bench-scale results, some microorganisms can use ECs as carbon or energy sources [65,76]. For example, carbamazepine was degraded as the sole carbon source by the bacterium *Gordonia polyophrenivorans* [78].

Emerging contaminants can also be degraded by photochemical transformation (a natural process) via direct absorption of solar radiation or indirect methods with photosensitized molecules [65]. These processes use solar energy to process organic ECs into other molecules by cleaving the covalent bonds, producing more biodegradable and hydratable compounds [79]. These degradation processes are influenced by several factors such as the type of compound, temperature, and pH [65,80,81]. Direct photochemical processes cleave the organic molecules by atmospheric surface photons and ultraviolet radiation. The process is highly dependent on the π-bond configuration of the ECs, as it affects the absorptive ability of the photons by the compound [65]. Conversely, indirect photochemical transformations use chromophores, which are excited by solar radiation, and pass them to the EC compound, thereby breaking the chemical bonds and degrading the molecule [65,79,81]. The efficiency of this process is dependent on the concentration of chromophores and their ability to react with organic ECs [79]. Emerging contaminants such as phenol can be degraded to form oxalic acid and methanoic acid via direct absorption of solar radiation or oxidation by hydroxyl radicals in water [82]. Cimetidine is photodegraded by reacting with a single oxygen [83], while diclofenac can be degraded by photo-cyclization and dichlorination-producing carbazole products [65]. Ibuprofen is degraded via direct photolysis and oxidation by hydroxyl radicals, producing isobutyl acetophenone, which is not typically considered to be easily biodegradable [65,84]. Notably, even after transformation or degradation, ECs and/or their intermediates can still have a detrimental effect on the aquatic system.

## 4. Microbial Biodegradation of Emerging Contaminants

In aquatic and terrestrial ecosystems, indigenous microorganisms may act as natural barriers to break down ECs. Degradation of ECs carried out by bacteria and fungi involves metabolic and catabolic reactions facilitated by different microbial enzymes through metabolism and co-metabolism processes, which can transform these organic molecules into low-complex molecules such as water (H_2_O) and carbon dioxide (CO_2_) [3,85,86]. Herein, we describe the metabolism and co-metabolism processes involved in the degradation of ECs and the environmental factors that affect their efficiency.

### 4.1. Bacterial Degradation of Emerging Contaminants

Bacteria can be divided, based on their metabolism, into two main groups: heterotrophic and autotrophic. Indeed, these groups have shown different strategies to metabolize or co-metabolize ECs. Heterotrophic bacteria degrade ECs metabolically by using them/ECs as the sole carbon source. For instance, bacterial species such as *Sphingomonas* Ibu-2 and *Rhizorhabus wittchi* MPO18 have demonstrated the capacity to degrade Ibuprofen, using it as the main carbon source under aerobic conditions [87,88]. Likewise, acetaminophen has also been reported to act as a carbon source for *Delftia tsuruhatensis* and *Pseudomonas aeruginosa* under aerobic conditions [89]. On the other hand, heterotrophs such as *Pseudomonas* sp. and *Azoarcus evansii* can carry out anaerobic degradation of phenylacetic acid [90]. Interestingly, *Azoarcus evansii* can also metabolize phenylacetic acid (Ibuprofen) under aerobic conditions. Many facultative microorganisms, such as *Pseudomonas* sp., *Bacillus* spp., and *Azoarcus evansii*, can shift their respiration mechanisms from aerobic to anaerobic conditions. This flexibility enables the bacteria to effectively degrade ECs under both environmental conditions [91]. Other microorganisms, such as sulphate-reducing microorganisms (i.e., autotrophs), which commonly consist of anaerobes, couple sulphate reduction to the metabolism of ECs [92]. It has been suggested that nitrate and sulphate can be alternative electron acceptors for the biodegradation of ECs in anaerobic sediments [93]. The ability of sulphate and nitrate reducers to co-metabolize organic contaminants while reducing nitrate or sulphate highlights their dual role in both detoxifying pollutants and cycling essential nutrients [94]. For instance, benzene oxidation to carbon dioxide linked to nitrate reduction was observed in enrichment cultures developed from groundwater microcosms [95]. Likewise, *Desulfovibrio* can oxidize benzene compounds such as benzyl alcohol, benzaldehyde, and 1,2,4-trihydroxybenzene with sulphate as the electron acceptor [96]. Interestingly, ECs such as antibiotics may interfere with a number of processes such as methanogenesis, sulphate reduction and nitrogen transformation in some bacteria [97]. In particular, Keen and Patrick (2013) [97] reported that, depending on their concentrations, the presence of antibiotics can inhibit or stimulate the activity of sulphate-reducing bacteria, thereby affecting the overall sulphate reduction process in contaminated environments.

Interestingly, heterotrophs can also co-metabolize ECs; however, the ECs need to reach a sufficient concentration threshold for the microbes to degrade them, without being toxic to the microbes [98]. Lui and co-workers reported the co-metabolism of imidacloprid (insecticide) by *Stenotrophomonans maltophilia* CGMCC 1.11788 using different carbon sources, affecting the fate of the ECs. This insecticide is known to be persistent (resistant) in plants, soil, and water [99]. When sucrose was used as the main carbon source, the imidacloprid was transformed into 5-hydroxy imidacloprid; however, when succinate was added, the imidacloprid contaminant was further converted to olefin imidacloprid, which can be further degraded to carbon dioxide via the olefin imidacloprid pathway [99]. The co-metabolisms of Ibuprofen and diclofenac can be facilitated by fumarate reductase in the presence of carbon sources such as fumarate and ethanol by the heterotrophic genus *Smithella* [100]. In addition, the ability of *P. putida* F1 to metabolize trichloroethylene (TCE) in the presence of toluene has been reported [101]. Several studies concluded that toluene enhances the ability of *P. putida* F1 to degrade TCE [102,103,104,105].

Atrazine, one of the most widely used pesticides in Southern Africa, can be co-metabolized by *Nocardioides* spp. using sodium citrate under aerobic conditions. However, the addition of sucrose as a carbon source reduced the rate of atrazine degradation, indicating that the concentration and type of additional substrates can significantly impact the co-metabolic processes [106]. Bacterial species such as *Pseudomonas* sp., *Rhodococcus rhodochorus*, *Acinetobacter* sp., *Bacillus* sp., and *Aerobactrium* sp. can hydrolyze atrazine to carbon dioxide and ammonia (Table 6) [69,70,75]. In a study under anaerobic conditions, atrazine was used as either the sole source of carbon or nitrogen. Under co-metabolic conditions with molasses as a co-substrate, atrazine was successfully removed under electron acceptor conditions. However, it is worth mentioning that the atrazine degradation efficiency was significantly higher under sulphate-reducing conditions than the nitrate-reducing conditions. Under sulphate-reducing conditions, atrazine removal was faster when molasses was used as a co-substrate. The metabolic analysis showed that atrazine was mineralized, and the major metabolites observed include hydroxy atrazine, cyanuric acid, chloride, and ammonium [107].

Unlike heterotrophs, autotrophic bacteria biodegrade ECs via non-specific monooxygenases instead of using ECs as the sole carbon source [98,108]. Autotrophic bacteria, such as ammonia oxidizers, facilitate the co-metabolism of ECs via the utilization of non-specific enzymes such as ammonium monooxygenases. Ammonium monooxygenases can degrade several ECs such as diclofenac, naproxen, clofibric acid, and bezafibrate under both aerobic and anaerobic conditions [98,109]. Under aerobic conditions, ECs are also oxidized by enzymes such as oxidoreductases (e.g., oxidases, peroxidases, oxygenases, dehydrogenases) and hydrolases (e.g., amidases, esterases) in the presence of carbon sources such as glucose [22,98]. For example, sulfamethoxazole co-metabolic oxidation by the horseradish peroxidase has been reported in cycling aerobic environments [22]. The co-metabolic biodegradation process for trichloroethylene (TCE) is also facilitated by nonspecific oxygenases such as methane monooxygenases, toluene monooxygenases, and phenol monooxygenase [84]. Methane monooxygenase uses methane as the primary substrate to co-metabolize TCE, producing carbon dioxide as the final product [84], facilitated by methane-oxidizing bacteria (methanotrophs). A study by Shukla and co demonstrated that a methanotrophic consortium could biodegrade TCE. Genes such as *pmoA* were identified as encoding for the methane monooxygenase (pMMO), which facilitates the co-metabolism of TCE with methane [86]. In anaerobic co-metabolism, ECs are reduced by reductive enzymes such as acetate kinase, reductive dehalogenase, and fumarate reductase [98,110]. For instance, Ibuprofen, diclofenac, and triclosan can be phosphorylated by acetate kinase during the acetogenesis process [22].

To further understand the two biodegradation metabolism processes and how they can be improved for higher EC removal efficiencies, different studies have focused on mapping out metabolic pathways and genes involved in the biodegradation of ECs. The genes are responsible for encoding enzymes that drive the metabolism pathways that can degrade ECs (Table 6). Genes are essential in understanding how these metabolic pathways function [111]. By using genome analysis, it is possible to identify these pathways [111]. Gedalanga et al. (2014) performed a study to identify the biomarker genes to predict the biodegradation of 1,4-dioxane. The authors targeted the multicomponent monooxygenase genes in *Pseudonocardia dioxanivroans* CB1190, which were used as a biomarker to identify the potential of 1,4-dioxane biodegradation. The results showed the upregulation of genes encoding for 1,4-dioxane monooxygenase, propane monooxygenase, alcohol dehydrogenase, and aldehyde dehydrogenase, which could indicate 1,4-dioxane degradation at concentrations ranging from 50–100 mg/L [112]. In this process, the enzymes, 1,4-dioxane monooxygenase and propane monooxygenase, initiate the breakdown of 1,4-dioxane by inserting an oxygen molecule into its ring structure. Thereafter, alcohol dehydrogenase oxidizes the resulting alcohols into aldehydes, which are further converted to carboxylic acids by the aldehyde dehydrogenase enzyme. These reactions lead to the removal and complete degradation of 1,4-dioxane, which highlights the use of genes as biomarkers of potential EC biodegradation. Gene identification can also be used to select microorganisms with the metabolic potential to biodegrade ECs. Other studies have also been able to identify genes involved in metabolism and co-metabolism processes of ECs, such as atrazine, sulfamethoxazole, toluene, and terbuthylazine, which can be used as biomarkers of potential EC biodegradation [113,114,115,116,117,118,119,120,121,122].

**Table 6:** Bacterial genes associated with the metabolism of various emerging contaminants.

Emerging Contaminants	Genes Associated with Metabolism	Enzymes	Bacterial Species	References
Atrazine	*trZn*, *atZA*, *atZB*, *atZC*	Atrazine chlorohydrolase, Hydroxyl-atrazine ethylamine hydrolase, N-isopropylammelide isopropyl amino hydrolase, allophanate hydrolase	*Acinetobacter* spp., *Bacillus* sp., *Microbacter* spp.	[113,123]
Acetaminophen	*amiB*, *ampD*	Amidases Atrazine chlorohydrolase Hydroxyl-atrazine ethylamine hydrolase	*Pseudomonas* spp.	[124]
Terbuthylazine	*trZn*, *atZA*, *atZB*, *atZC* *atZA*, *atZB*, *atZC*	N-isopropylammelide isopropyl amino hydrolase, allophanate hydrolase	*Pseudomonas putida*	[117,124]
Sulfamethoxazole	*amoA*, *amoB*, *amoC*, *hao*, *nxrAB*	Ammonia monooxygenase, hydroxylamine oxidoreductase,	*Nitromonas*, *Nitrospira*, *Nitrocsococcus*	[98,125]
Erythromycin Fluoxetine Roxithromycin	*NIR*, *NOD*, *pMMO*, *MDH*, *mtdB*, *FDH*	Nitric oxide reductase, methane monooxygenase, methane dehydrogenase	(*Candidatus* Methylomirabilis species—*Ca*. M. oxyfera; *Ca*. M. *sinica; Ca*. M. *lanthanidiphila*)	[24]
Trichloroethylene	*MmoBCDXYZ (cluster)*	Methane monooxygenase cluster	*Methylosinus trichosporium*	[85]
Carbamazepine	*bphC*	Biphenyl-2,3-dioxygenases	*P. xenovorans*, *Pseudomonas* spp.	[121,122,126]

### 4.2. Fungal Biodegradation of Emerging Contaminants

Fungi play a crucial role in the direct degradation (or metabolism) of ECs, and their genomic capabilities significantly contribute to this process. ECs encompass a wider range of different compounds, thus degradation of these is not confined to one fungal taxon (Table 7). The genomic analysis of various fungal species has revealed a diverse array of genes and metabolic pathways that facilitate the breakdown of complex pollutants, including hydrocarbons, polycyclic aromatic hydrocarbons (PAHs), and pesticides [18]. Fungi can perform degradation of ECs reverently under aerobic conditions. Although the degradation under anaerobic conditions is also plausible, the exact mechanism under anoxic conditions is not well understood. Briefly, in anaerobic PAH degradation, radicals or inorganic compounds seem to replace oxygen as electron acceptors, with enzymes catalyzing PAH activation and breakdown into simpler compounds like carbon dioxide and water through processes like beta-oxidation [127].

A key factor in aerobic fungal metabolism for degrading ECs is the presence of specific enzymes (e.g., laccase, dioxygenase, and peroxidase) (Table 7) [18]. For example, the genome of *Aspergillus sydowii* has been shown to encode peroxidases, such as lignin peroxidase and manganese peroxidase, which are crucial for the oxidation of PAHs under aerobic conditions [128]. These enzymes enable the fungus to initiate the degradation of complex hydrocarbons, transforming them into less harmful compounds. Similarly, *Lasiodiplodia theobromae* has demonstrated enhanced lignin peroxidase activity during the degradation of benzo[a]pyrene, a significant PAH, indicating the importance of these enzymes in the metabolic pathways of fungi involved in bioremediation [128]. The genomic diversity among fungal species also plays a significant role in their bioremediation capabilities. For instance, studies have shown that indigenous fungi can adapt to hydrocarbon-contaminated environments through genetic modifications that enhance their ability to catabolize xenobiotic chemicals [129]. This adaptability is often linked to the presence of specific gene families that encode for carbohydrate-active enzymes (CAZymes), which are essential for the degradation of complex organic materials [130]. The comparative analysis of CAZymes in different fungal taxa has revealed significant variations, suggesting that certain fungi are better equipped to degrade specific contaminants based on their genomic makeup [130]. Cytochrome P450 monooxygenases also play a critical role in fungal degradation processes. These intracellular enzymes are essential for the oxidative metabolism of a wide variety of environmental pollutants, including aromatic compounds and pesticides [131]. Notably, the white-rot fungus *Phanerochaete chrysosporium* harbors around 150 P450 monooxygenase genes in its genome [132]. This fungus has been shown to utilize two specific cytochrome P450 isozymes to degrade four neonicotinoids: acetamiprid, clothianidin, imidacloprid, and thiacloprid [133].

**Table 7:** Fungal genes associated with the metabolism of various emerging contaminants.

Emerging Contaminants	Genes Associated with Biodegradation	Enzymes	Fungi	References
Fipronil	*cyp51F1*	Cyp50 moassociatednoxygenase	*Trametes versicolor*	[18,134]
Bisphenol A	*p2ox*	Manganese peroxidase	*Phanerochaete chrysosporium*	[18,135]
Imidacloprid	*cyp51F1*	Cyp50 monooxygenase	*Trametes versicolor*	[18,136]
Carbamezapine	*lcca*	Laccase	*Trametes versicolor*	[18,135]
Diclofenac	*lipB*	Lignin peroxidase	*Phanerochaete chrysosporium*	[18,135]
Acetaminophen	*lcca*	Laccase	*Bjerkandera spp.*	[18,137]
Atrazine	*lcca*	Laccase	*Trametes versicolor*	[18,138]

## 5. Effect of Biological and Chemical Factors on EC Biodegradation

Indigenous microorganisms might serve as natural barriers by breaking down emerging contaminants (ECs). However, biological and chemical factors such as seasonal variations, especially dry and wet seasons, the availability of oxygen, nutrients, EC concentrations, and the type of microorganisms within aquatic environments may boost or hinder the effectiveness of EC biodegradation. Here, we highlight the factors that, in our opinion, could influence ECs’ biodegradation.

### 5.1. Seasonal Variation

The seasonal variation (including temperature fluctuations and precipitation patterns) can significantly influence the composition and structure of microbial communities. For instance, Liu et al. (2022) noted that the relative abundance of certain bacterial groups decreased during the wet season, potentially affecting the degradation of organic compounds [139]. Similarly, seasonal shifts in microbial community composition can alter the rates of carbon and nitrogen cycling, thereby impacting the overall ecosystem productivity and health [140]. Consequently, it seems reasonable to think that the seasonal shift might affect the biodegradation of ECs. Indeed, the increase in precipitation during the wet season leads to higher water levels, which can enhance the transport and dilution of contaminants. This dilution effect might facilitate the bioavailability of ECs, making them more accessible to microbial communities for degradation. However, to the best of our knowledge, no studies have been reported that demonstrate this process in the context of EC biodegradation. Temperature also plays a critical role in these seasonal dynamics. In South Africa, the wet season often brings warmer temperatures, which can enhance microbial metabolic rates and, consequently, the biodegradation of contaminants. Conversely, cooler temperatures during the dry season can slow down microbial processes, which may lead to decreased biodegradation rates [141].

### 5.2. Microbial Structure

Although this was not discussed previously, the microbial lifestyle could influence the EC biodegradation. For instance, planktonic cells of *Candida tropicalis* can degrade certain contaminants such as phenol rapidly under ideal conditions [142]. Likewise, Sekhar et al. (2016) found that the specific degradation rates of the contaminant 2,6-dichlorobenzamide (BAM) were significantly higher in planktonic cells than in sessile cells, with rates approximately 100-fold greater for suspended fresh cells compared with sessile cells [143]. This difference can be attributed to substrate mass-transfer limitations within biofilms, which can restrict the diffusion of contaminants into the biofilm matrix [143]. However, it is essential to note that while planktonic cells may exhibit higher degradation rates under certain conditions, such as concentrations of the ECs and long-term exposure to ECs and simultaneous exposure to different ECs [144], biofilm cells often demonstrate greater resilience and long-term stability in contaminated environments. For instance, Zhao et al. (2016) reported that biofilm-associated cells of *Pseudomonas stutzeri* T102 showed enhanced degradation activity for naphthalene compared with planktonic cells, indicating that biofilms can be more effective in degrading specific ECs [145]. The ability of biofilms to maintain higher microbial biomass and facilitate intercellular communication through signaling molecules further contributes to their enhanced degradation capabilities [146]. In addition, another factor that could influence microbial interactions with ECs is their solubility. Highly soluble ECs are more likely to interact with planktonic cells, while less soluble ECs will be associated with biofilms attached to surfaces like sediments. Consequently, both planktonic and sessile cells are expected to contribute, to varying degrees, to the degradation of ECs in aquatic systems.

### 5.3. Oxygen Requirements

The concentration of oxygen dictates whether aerobic or anaerobic conditions prevail, which can affect the biodegradation of ECs in aquatic environments [147,148,149]. Aerobic biodegradation is generally considered more favorable for the degradation of many ECs [148,150]. This is because aerobic biodegradation is considered to be energetically favorable compared to strict anaerobic conditions [148]. While aerobic conditions are often preferred, anaerobic conditions can also promote higher biodegradation efficacy of ECs, such as triclosan [151], naphthalene [152], sulfamethoxazole [153], carbamazepine [154], and imazosulfuron [155], among others. Ghattas et al. (2017) reported that certain aerobically recalcitrant contaminants could still be biodegraded under anaerobic conditions, indicating that anaerobic processes can be effective under specific circumstances [148].

Another aspect we should take into consideration is the formation of intermediate compounds. The production of intermediate compounds during the biodegradation of ECs is influenced by the metabolic pathways utilized by microorganisms under aerobic and anaerobic conditions [155,156]. Generally, aerobic biodegradation tends to produce fewer intermediate compounds compared with anaerobic processes, which often result in a more complex array of metabolites [148,157,158,159]. Aerobic biodegradation typically involves the complete oxidation of organic contaminants, leading to the formation of carbon dioxide and water as final products. In contrast, anaerobic biodegradation processes, such as reductive dichlorination, often result in the accumulation of various intermediate compounds [95,148,158,159,160]. For example, the degradation of aromatic compounds under anaerobic conditions can lead to the formation of intermediates such as catechols and other substituted aromatic compounds, which are not typically produced during aerobic degradation [148,161,162].

### 5.4. Metabolisms, Co-Metabolisms, and Synergisms

Usually, the co-metabolism process is considered the main biodegradation mechanism for ECs’ degradation and/or removal [22,24,85]. However, metabolic processes tend to promote more efficient and complete degradation of contaminants compared with co-metabolism processes [98,163,164]. This is because metabolism pathways are specifically tailored for utilizing the contaminant as the main carbon and energy source, leading to higher degradation rates and more complete mineralization. In contrast, co-metabolism may result in partial degradation or transformation of the contaminant, which can sometimes lead to the formation of intermediate products that may still be harmful [98,122,165,166,167]. However, co-metabolism can be advantageous when contaminants are present in low concentrations or when the microorganisms cannot utilize the contaminants directly [22,23,24]. In such cases, co-metabolism can mitigate pollution without requiring the microorganisms to adapt specifically to the contaminant. Due to the different complex microbial populations found in real environmental systems and low concentrations of ECs in natural environments, both metabolism and co-metabolism processes may co-exist [98,116].

Moreover, the synergy between species and kingdoms could catalyze the degradation of ECs. Several studies have demonstrated that fungal consortia, as opposed to individual strains, can effectively degrade certain pesticides. For instance, a consortium of five fungal isolates (three from the genus *Pleurotus*, one closely related to *Coriolopsis*, and one unidentified) exhibited greater efficiency in degrading the pesticides diazinon and methomyl compared with single isolates [168]. A similar trend was observed in consortia, combining fungi (*Mortierella* LEJ702) and bacteria (*Variovorax* SRS16 and *A. globiformis* D47) for the degradation of diuron [169]. Additionally, *Mortierella* spp. LEJ702 enhanced the breakdown of the herbicide 2,6-dichlorobenzamide mutually with *Aminobacter* spp. MSH1 [170]. However, the fungal role in these processes may not have been involved in the direct degradation of the compounds but rather in aiding bacterial dispersal through fungal hyphae or translocating the contaminants.

From the aforementioned factors and their influence on the EC biodegradation, it is evident that the efficiency of the biodegradation process is not linear but rather is influenced by a complex interaction of biological and chemical factors. Therefore, a deeper understanding of these factors is necessary to optimize the biodegradation process in real-world settings. Seasonal variability consisting of changes in temperature and precipitation patterns has a great influence on the microbial dynamics, which in turn has an influence on the biodegradation of ECs [139]. These microbial shifts may similarly influence EC degradation capacities, although direct evidence remains sparse. While it has been hypothesized that higher precipitation during wet seasons could enhance ECs’ bioavailability via dilution and transport mechanisms, this remains largely speculative because empirical data linking seasonal hydrological changes to EC biodegradation is lacking [141]. Furthermore, temperature is a critical, yet often disregarded, factor in these systems. Thus, there is a need for targeted research assessing the combined effects of temperature, hydrodynamics, and microbial adaptation on ECs’ biodegradation across seasons [141].

The structural form of microbial communities, whether planktonic or biofilm-associated, significantly impacts ECs’ biodegradation potential [143,144]. While planktonic cells often exhibit rapid degradation rates under controlled laboratory conditions, these findings do not always translate to natural settings where biofilms dominate. Biofilms are generally characterized by augmented resilience, long-term stability, and cooperative metabolic interactions, often resulting in superior degradation of specific ECs under environmental stressors [145,146,147]. However, this difference is not absolute. Factors such as EC solubility and environmental concentrations influence whether planktonic or sessile microorganisms dominate EC degradation processes. Highly soluble ECs are more accessible to planktonic cells, whereas less soluble compounds may preferentially accumulate within biofilms. Importantly, these relationships are dynamic and context-dependent, indicating that both microbial forms likely contribute synergistically in complex aquatic systems. Future studies should prioritize integrating both microbial lifestyles within EC degradation models to better reflect environmental realities.

Oxygen levels may serve as a primary determinant of the biodegradation pathways utilized by microbial communities [148,149,150]. While aerobic degradation is widely regarded as more energetically favorable and effective for many ECs [149,150,151], emerging evidence highlights the unrecognized role of anaerobic processes in degrading traditionally recalcitrant contaminants [149]. Compounds such as triclosan and carbamazepine, often resistant under aerobic conditions, have been shown to undergo degradation under anaerobic settings [152,155]. Nevertheless, a critical outcome of this process is the formation of intermediate compounds, which are often more diverse and potentially toxic under anaerobic conditions due to incomplete mineralization and reductive pathways [148,156,157]. Typically, aerobic degradation tends to result in complete oxidation to innocuous end-products like CO_2_ and H_2_O. On the other hand, anaerobic systems frequently accumulate intermediates and other aromatic derivatives. This raises important questions regarding the trade-offs between degradation efficiency and metabolite toxicity, which require more detailed investigation, particularly within mixed redox environments that typify natural aquatic systems [148,158,159].

The mechanisms by which ECs are degraded, whether via direct metabolism or co-metabolism, also dictate the overall degradation efficiency and completeness [22,24,85]. While metabolic degradation pathways offer more efficient and complete breakdown due to the direct use of contaminants as carbon or energy sources, co-metabolic processes are often more prevalent in real-world conditions where EC concentrations are low [98,122,165,166,167]. Notably, co-metabolism plays a valuable role in mitigating EC pollution, especially in complex ecosystems where microbial populations may not be specifically adapted to utilize contaminants as primary substrates [22,23,163]. The co-existence of metabolic and co-metabolic processes in aquatic environments underscores the complexity of biodegradation under environmental conditions, where multiple mechanisms may operate concurrently [98,116]. Additionally, interspecies and inter-kingdom synergisms have emerged as potent drivers of EC degradation. Fungal–bacterial consortia, in particular, have demonstrated superior degradation performance for certain pesticides and herbicides compared with single species [168,169,170]. These synergies may not only enhance degradation rates but also improve the spatial distribution of bacteria, potentially expanding the functional niche for EC biodegradation. However, such systems remain underexplored outside of laboratory settings, warranting field-based studies to validate these promising interactions. In sum, the biodegradation of ECs in aquatic systems is a highly multifaceted process shaped by seasonal, microbial, chemical, and ecological factors. While laboratory studies have provided valuable mechanistic insights, there remains a critical knowledge gap regarding how these factors interact under environmental conditions. Future research should prioritize integrated, field-based studies that account for the simultaneous effects of hydrological dynamics, microbial ecology, and contaminant chemistry. Such approaches will be essential for developing robust, nature-based solutions for the management of EC pollution in aquatic ecosystems.

## 6. Effects of Emerging Contaminants on the Microbiome

Emerging contaminants pose a threat to microbial communities in the environment (Table 8). These compounds are often not easily removed by conventional wastewater treatment processes and tend to persist at low concentrations in soil and water as well as within human hosts. High concentrations of ECs may induce changes in the microbiome (Table 8). ECs seem to increase microbial richness but decrease microbial diversity [171]. They can change the overall diversity of microbial communities and their functionality, which in turn changes the ecosystems’ functionality and biodiversity of the aquatic environment [5]. Emerging contaminants have been linked to changes in respiration rate, extracellular polymer substances (EPSs) production, and gene expression [5]. They can also interact with environmental biofilms’ EPSs via uptake directly from the water, absorption into the bacterial surface by binding to specific sites, and siderophores [5]. Emerging contaminants such as antibiotics can also be trapped in these biofilms [172]. A study by Prioa et al. (2013) reported that high concentrations of quinolones and sulfonamides, such as sulfamethoxazole (234 ng/L), decreased the bacterial biomass in biofilms by inhibiting the production of dihydrofolic acid, which is needed for bacterial survival [173,174]. Even so, microbial cultures have evolved resistant mechanisms that allow them to survive under these adverse conditions. For instance, the presence of ECs such as sulfadiazine and ciprofloxacin promoted high enzymatic activity, producing more EPSs, which leads to increased biomass [175]. Moreover, in the presence of ECs such as sulfadiazine and ciprofloxacin, bacterial species such as *Hyphomicrobium* and *Sphingomonas* could express antibiotic-resistance genes such as class I integrons (*intI1*), which resulted in a microbial shift and an increase in biomass [175]. Also, polycyclic aromatic compounds such as naphthalene and phenanthrene accelerate the propagation of antimicrobial resistance genes such as class I integrons (*intI1*), sulfonamide resistance genes (*sul1*)*,* and aminoglycoside resistance genes (*aadA2*).

## 7. Conclusions

Emerging contaminants (ECs) are a public concern in many countries because of their potential negative impacts on terrestrial and aquatic ecosystems and their transmission to humans through the food chain, especially by bioaccumulation and biomagnification in animals and plants. Indigenous bacteria and fungi have shown the capability to metabolize and co-metabolize ECs, which might act as natural barriers to degrading these organic pollutants. However, the complete removal of these ECs seems to rely on biological and chemical factors such as seasonal variations, the availability of oxygen and nutrients, EC concentrations, and the type of microorganisms. In aquatic ecosystems, seasonal variation will regulate the concentration of ECs in solution. Even though the chemical properties, such as the solubility of ECs, will influence their fate, the increase in precipitation and water levels in the lagoons and rivers can enhance the transport and dilution of contaminants. Unfortunately, the bioavailability of ECs will not ensure their complete biodegradation. Wet and dry seasons can shift the conditions in aquatic ecosystems. Temperature, oxygen, nutrient, and EC concentrations may change during the wet and dry seasons, affecting and changing the microbial diversity and activity, which will have an enormous impact on the ECs’ complete biodegradation. The oxidative microbial process, under mesophilic conditions, seems to biomineralize ECs into carbon dioxide and water fully. These conditions are more plausible in South Africa during the wet season (summer). Therefore, significant microbial activity and higher removal of ECs would be expected due to the ideal temperature, increased oxygen concentrations, and bioavailability of nutrients and ECs. However, the EC concentration for triggering microbial metabolic activities is uncertain. Due to the low EC concentration and the bioavailability of other nutrients that often predominate in aquatic ecosystems during the wet season, EC degradation might be feasible through co-metabolism. Regrettably, co-metabolism leads to the generation of intermediate compounds, some of which are still harmful even for microorganisms that carry on biodegradation. The synergistic activity of bacteria and fungi might overcome this issue and contribute to the complete degradation of ECs. However, to date, no studies have addressed the proper monitoring of ECs and microbial activity (in planktonic and sessile cells) in aquatic systems that may support this hypothesis. Therefore, an integrated study where all these parameters are included might provide a holistic and realistic perspective of the ECs’ biodegradation in aquatic environments. This holistic approach should be able to cover metabolism and co-metabolism, which may co-exist in the natural environment, to further increase the efficiency of microbial treatment of ECs. More studies should focus on determining optimal concentrations at which the microbes use these ECs for metabolic purposes. This will ensure that large-scale use of the biodegradation process can be optimized to remove higher concentrations of ECs from the aquatic environment. Further studies into the mechanisms used by enzymes found in microbes to remove ECs from the environment. This will allow for the designing of consortiums that can be used for bioaugmentation in natural environments, ensuring safer and greener methods for EC removal.

## Figures and Tables

**Table 1:** Common emerging pharmaceutical contaminants.

Compound	Application	Chemical Formula	Molecular Weight (g/mol)	pKa	LogK_ow_	Solubility (mg/L)	References
Acetaminophen	Analgesic	C_8_H_9_NO_2_	151.16	9.38	0.46	14 × 10^3^	[29,44]
Atenolol	β-Blocker	C_14_H_22_N_2_O_3_	266.34	9.6	0.16	13 × 10^3^	[29,44]
Carbamazepine	Anti-epileptic	C_15_H_12_N_2_O	236.27	13.9	2.45	18	[29,44]
Diclofenac	Anti-inflammatory	C_14_H_11_Cl_12_NO_2_	296.14	4.15	4.51	4.47	[29,44]
Ibuprofen	Anti-inflammatory	C_13_H_18_O_2_	206.28	4.91	3.97	21	[29,44]
Ranitidine	Antihistamine	C_13_H_22_N_4_O_3_S	314.40	8.4	0.99	1000	[29,42]
Sulfamethoxazole	Antibiotic	C_12_H_14_N_4_O_2_S	278.33	7.59	0.14	610	[29,44]
Salicylic acid	Analgesic	C_7_H_6_O_3_	138.12	2.97; 13.6	2.26	2240	[29]

**Table 2:** Common emerging personal care products’ contaminants.

Compound	Application	Chemical Formula	Molecular Weight (g/mol)	pKa	LogK_ow_	Solubility (mg/L)	References
Climbazole	Antifungal	C_15_H_17_ClN_2_O_2_	292.76	-	-	-	[46]
Ethinylestradiol	Hormone	C_20_H_24_O_2_	296.40	1.70	-	1.70	[45,46]
Galaxolide	Fragrances	C_18_H_26_O	258.40	-	-	1.75	[29,46]
Tonalide	Fragrances	C_18_H_26_O	258.4	-	-	1.25	[29,46]
Triclosan	Anti-microbial	C_12_H_7_Cl_3_O_2_	289.53	7.9	4.76	10	[29,46]

**Table 3:** Common emerging per- and poly-fluroalkyl substances contaminants.

Compound	Application	Chemical Formula	Molecular Weight (g/mol)	pKa	LogK_ow_	Solubility (mg/L)	References
Trifluoroacetic acid	Adhesive and sealant	C_2_HF_3_O_2_	111.02	0.52	-	-	[55]
Perfluorobutanoic acid	Firefighting foams	C_4_HF_9_O_3_S	300.10	-	-	-	[54,55]
Perfluorohexane phosphonic acid	Food packaging	C_6_HF_13_O_3_S	400.11	−3.45	-	-	[54,55]
Hexafluoropropylene oxide trimer acid	Industrial organofluorine chemistry	C_3_F_6_O	166.02	-	-	-	[54,55]

**Table 4:** Common emerging pesticides contaminants.

Compound	Application	Chemical Formula	Molecular Weight (g/mol)	pKa	LogK_ow_	Solubility (mg/L)	References
Organochlorides (DDT)	Insecticide	C_14_H_9_Cl_15_	702.00	-	6.91	0.006	[57,58]
Organophosphates (Azinphos-methyl)	Insecticide	C_10_H_12_N_3_O_2_PS_2_	300.00	-	2.96	28	[57,58]
Carbamates (Aldicarb)	Insecticide	C_7_H_14_N_2_O_2_S	190.00	-	1.15	4930	[57,58]
Pyrethroids (Acrinathrin)	Insecticide	C_26_H_21_F_6_NO_5_	541.00	-	5.6	<0.02	[57,58]

**Table 5:** Maximum concentrations of emerging contaminants detected in South African waters.

Emerging Contaminant	Source	Concentration (ng/L)	References
Atrazine	Surface water	1237	[64]
Terbutylazine	Surface water	1969	[64]
Atenolol	Groundwater	25,900	[64]
Sulfamethoxazole	Wastewater	34,500	[64]
Nevirapine	Surface water	1480	[66]
Zidovudine	Surface water	973	[66]
Lamivudine	Surface water	242	[66]
Stavudine	Surface water	778	[66]

**Table 8:** The impact of emerging contaminants on the microbial community and the microbial community’s response to the exposure.

Emerging Contaminant	Impact	Microbial Response	Reference
Sulfamethoxazole	Decrease the bacterial biomass in biofilms by inhibiting the production of dihydrofolic acid.	The microbial cells produce more EPSs to act as a protective barrier against the compound.	[173,174,176]
Oxytetracycline	Increase in microbial biomass carbon, which led to an increase in nitrification potential and dehydrogenase activity.	The microbial community adapts to the metabolic shift, resulting in a microbial shift to a community in favor of members that are more tolerant to the ECs and capable of EC biodegradation.	[177,178]
Carbamazepine	Disruption in the microbial diversity within the community leads to the selection of antibiotic-resistant genes.	The microbial community acquires antibiotic-resistant genes as a result of horizontal gene transfer.	[179,180,181]
Amphetamine	Amphetamine disrupts the photosynthetic process, which can cause a decrease in the oxygen levels, shifting to a more anaerobic community and eventually decreases the bacterial composition.	The microbial community may shift towards anaerobic biodegraders with enhanced expression of detoxification enzymes.	[182]
Diphenhydramine	Inhibit the growth and proliferation of bacteria, which can lead to a decline in aerobic fast-oxidizing microbes.	Bacterial species adapt to diphenhydramine concentrations in wastewater and demethylate diphenhydramine into *N*-desmethyl diphenhydramine.	[183,184]

## Data Availability

No new data were created or analyzed in this study. Data sharing is not applicable to this article.
